# MiR-137 promotes TLR4/NF-κB pathway activity through targeting KDM4A, inhibits osteogenic differentiation of human bone marrow mesenchymal stem cells and aggravates osteoporosis

**DOI:** 10.1186/s13018-023-03918-y

**Published:** 2023-06-21

**Authors:** Ying-feng Yu, Pei-quan Yao, Zhi-kun Wang, Wen-wei Xie

**Affiliations:** Department of Orthopedics, Songshan Lake Central Hospital of Dongguan City, Dongguan, Guangdong China

**Keywords:** Osteoporosis, miR-137, TLR4/NF-κB, KDM4A, Human bone marrow mesenchymal stem cells (hBMSCs)

## Abstract

**Purpose:**

As the global population ages rapidly, osteoporotic fractures have become an important public health problem. Previous studies have suggested that miR-137 is involved in the regulation of bone formation, but its specific regulatory mechanism remains unclear. In this study, we aimed to explore the expression, role, and regulatory mechanism of miR-137 in the osteogenic differentiation of human bone marrow mesenchymal stem cells (hBMSCs).

**Methods:**

hBMSCs were induced into osteoblasts at first, and the expression level of miR-137 at different time points was detected. After knockdown and overexpression of miR-137, the effect of miR-137 on the osteogenic differentiation of hBMSCs was examined through alkaline phosphatase (ALP) staining and Alizarin Red staining. Western blotting was performed to detect the expression of runt-related transcription factor 2 (Runx2), osteocalcin (OCN), and toll-like receptor 4 (TLR4)/nuclear factor-κB (NF-κB) pathway. Bioinformatics websites were used to predict the target binding sites for miR-137 and KDM4A, and the results were validated using luciferase reporter gene experiments. Moreover, the ALP activity, calcium nodule formation, and activation of Runx2, OCN, and TLR4/NF-κB pathways were observed after knockdown of KDM4A.

**Results:**

The expression of miR-137 decreased during osteogenic differentiation. Knockdown of miR-137 expression increased the osteogenic ability of hBMSCs, while overexpression of it weakened the ability. Through the activation of the TLR4/NF-κB pathway, miR-137 inhibited osteogenic differentiation. KDM4A was identified as a predicted target gene of miR-137. After knocking down KDM4A expression, the osteogenic ability of hBMSCs was diminished, and the TLR4/NF-κB pathway was activated. Furthermore, the osteogenic ability of hBMSCs was partially restored and the activation level of TLR4/NF-κB was reduced after miR-137 knockdown.

**Conclusion:**

MiR-137 enhances the activity of the TLR4/NF-κB pathway by targeting KDM4A, thereby inhibiting the osteogenic differentiation of hBMSCs and exacerbating osteoporosis.

**Supplementary Information:**

The online version contains supplementary material available at 10.1186/s13018-023-03918-y.

## Introduction

Osteoporosis is a chronic metabolic bone disease that decreases bone mass and bone strength as well as increases the risk of fractures through altering the distribution of bone microstructure. Osteoporosis is brought on by various factors, including genetics, environmental factors, and immune abnormalities [1]. Currently, osteoporosis has gradually become a high-risk disease that not only severely affects the quality of life of the elderly but also places a heavy financial and psychological burden on families and society. Osteoporotic fractures have emerged as a significant public health issue worldwide due to the rapidly aging population [2, 3].

In several studies, 13 miRNAs, 70 lncRNAs, and 260 circRNAs are found to be differentially expressed in patients with postmenopausal osteoporosis compared with the healthy controls [3, 4]. As a type of non-coding RNAs (ncRNAs) with roughly 18–25 nucleotides, microRNAs (miRNAs) can bind to the 3′ untranslated region of their target RNAs and prevent those RNAs from being translated [5–8]. Multiple studies have revealed the therapeutic potential of miRNAs in arthritis and tendon injury [9–12]. And interestingly, among the miRNAs, miR-137 is essential for bone development, bone morphogenesis and bone formation after fractures [13]. The first report about miR-137 can be traced back to 2002, which aimed to identify functional miRNAs and their tissue-specific expression patterns in mammals [14]. MiR-137 can down-regulate the LSD1 gene expression through cooperation with the transcriptional co-repressor TLX, thereby promoting cell differentiation while inhibiting the proliferation of embryonic neural stem cells [15]. On the other hand, miR-137 can promote neural differentiation of various stem cells, including neural stem cells from adult mice, mouse oligodendroglioma-derived stem cells, and human glioblastoma multiforme-derived stem cells.

A considerable amount of studies, including the ones mentioned above, have indicated that miR-137 plays an essential role in regulating proliferation and differentiation [16]. MiR-137 can alter bone density in osteoporotic model rats by negatively regulating runt-related transcription factor 2 (Runx2), as demonstrated by relevant studies, but the mechanism of the regulation remains unclear. In spinal cord injury mice, circRNA3616 can inhibit toll-like receptor 4 (TLR4)/nuclear factor-κB (NF-κB) activity by absorbing miR-137 and reduce inflammation and apoptosis [17]. Besides, miR-137 can specifically inhibit TCF4 through the AMPK/NF-κB signaling pathway to stop the progression of osteoarthritis [18]. It makes sense to assume that miR-137, via a particular medium, may promote the activation of the TLR4/NF-κB pathway and exacerbate the progression of osteoporosis. Lysine-specific demethylase 4A (KDM4A) is a new epigenetic regulator of osteoblast and adipocyte differentiation [19]. According to published research, miR-137 can target KDM4A mRNA in the process of ras-induced aging [20]. In rats with spinal cord injury, miR-137 can encourage the differentiation of oligodendrocyte precursor cells and the myelination of axons via targeting KDM4A [21]. Therefore, we speculate that miR-137 may activate TLR4/NF-κB pathway by targeting KDM4A, thereby inhibiting the osteogenic differentiation of mesenchymal stem cells and worsening osteoporosis.

## Materials and methods

### Cell culture and osteogenic induction

Human bone marrow mesenchymal stem cells (hBMSCs) (HUXMA-01001) were purchased from Cyagen Biosciences (Soochow, China). In the standard culture medium (CM) group, the cells were cultured in Dulbecco's Modified Eagle Medium (DMEM, Gibco, USA) supplemented with 100 U/mL penicillin, 10% fetal bovine serum (FBS, Gibco), and 100 μg/mL streptomycin. The cells were incubated at 37 °C in a humidified atmosphere of 5% CO_2_. For osteogenic induction, hBMSCs were cultured in osteogenic medium (OM), which consisted of Alpha Modified Eagle's Medium (α-MEM) containing 10% FBS, 1% penicillin–streptomycin, 50 ng/mL ascorbic acid, 10 mmol/mL *β*-glycerophosphate, and 4 ng/mL dexamethasone (Thermo Fisher, USA) [22]. The medium was changed every 3 days, and the cells were cultured for 14 days for an observation of osteogenic differentiation by alkaline phosphatase (ALP) staining and Alizarin Red staining.

### Transient transfection

When the cell fusion rate reached approximately 60%, the medium was replaced with a fresh one and the cells were placed in an incubator. The transfection mixture was prepared by mixing 117.5 μL of serum-free α-MEM and 7.5 μL of Lipofectamine 3000 to make mixture A, and mixing 117.5 μL of serum-free α-MEM and 7.5 μL of miR-137 mimic/negative control or 110 μL of serum-free α-MEM and 15 μL of miR-137 inhibitor/negative control or KDM4A siRNA/negative control to make mixture B. The two mixtures were then combined and incubated at room temperature for 15 min before being added to the cells in each treatment group.

### Quantitative real-time polymerase chain reaction (qRT-PCR)

Total RNA was extracted from the cells using TRIZOL reagent (Invitrogen, Waltham, MA, USA), and cDNA synthesis was performed using the PrimeScript RT reagent kit (Takara, Japan). The expression levels of target genes were detected using SYBR Green Master Mix (Roche, Branchburg, NJ, USA). qPCR reaction was performed on a 7500 real-time PCR system (Applied Biosystems, Foster City, CA), with the reaction parameters as follows: 4 min at 95 °C for pre-denaturation, 10 s at 95 °C for denaturation following a cycle of 40 times, 30 s at 52 °C for annealing, 30 s at 72 °C for extension, and 10 min at 72 °C for final extension. The primer sequences used are as follows: the forward primer for miR-137 was 5′-GCGCGCTTATTGCTTAAGAATAC-3′, and the reverse primer was 5′-GTGCAGGGTCCGAGGT-3′; the forward primer for KDM4A was 5′-GCCGCTAGAAGTTTCAGTGAG-3′, and the reverse primer was 5′-GCGTCCCTTGGACTTCTTATT-3′; the forward primer for GAPDH was 5′-GTATAATGAGAAGCCAGACCAT-3′, and the reverse primer was 5′-ACAGCTTCTCAAGTCT-3′. The qRT-PCR data was normalized using the GAPDH expression as an internal control. The relative gene expression was calculated using the 2^−ΔΔCt^ method.

### Alkaline phosphatase (ALP) staining and Alizarin Red staining

After transfection with miR-137 mimics or inhibitors, early mineralization was observed by the BCIP/NBT ALP staining kit (Beyotime) following the manufacturer's instructions. The cells were fixed with 4% paraformaldehyde (Beyotime) for 10 min, washed three times with PBS, and then incubated in BCIP/NBT staining solution at 37 °C in the dark for 30 min. After two washes with distilled water, the cells were observed and photographed, and the ALP concentration was quantified. Late-stage mineralization was determined under the instructions of the osteoblast-mineralized nodule staining kit (Beyotime). After fixation with 4% paraformaldehyde for 10 min, the cells were washed three times with PBS and stained with Alizarin Red for 10 min. Next, the cells were washed again with distilled water, followed by microscopy and photography. The calcium nodule level was quantitatively analyzed at last.

### Bioinformatics prediction

After the analysis of bioinformatics websites such as Targetscan, miRWalk, miRBase, and Starbase, the results from each website were intersected to analyze the base complementarity with miR-137 3′-UTR region and the expression abundance of the target mRNA.

### Dual-luciferase reporter assay

293 T cells and target plasmids in 96-well plates were prepared for transfection. When the cell density reached 50–70%, 10 μL of α-MEM, 0.16 μg of mRNA mutant target plasmid and 5 pmol of miR-137 mimics/mimics NC were mixed and then cultured at room temperature (solution A). Subsequently, 10 μL of α-MEM was combined with 0.3 μL of transfection reagent (solution B) at room temperature for 5 min. Using a pipette, the two solutions were mixed and left to stand for 20 min. After replacement of the culture medium, the cells were added to the resulting mixture and later cultured in an incubator at 37 °C and 5% CO_2_ for 48 h before collection.

### Western blotting analysis

The collected cells were washed with PBS and lysed in RIPA lysis buffer (Beyotime) containing 2% protease inhibitor for 30 min. The protein concentration was determined using the BCA kit (Beyotime). Specifically, 20 μg of protein was boiled with 1 × loading buffer, separated by sodium dodecyl sulfate–polyacrylamide gel electrophoresis (SDS-PAGE), and then transferred to polyvinylidene fluoride (PVDF) membranes. Following centrifugation at 12,000 × g for 20 min, the separated protein was injected into 10% SDS-PAGE and then transferred to PVDF membranes. After being blocked with 5% skim milk for 1–3 h, the membranes were incubated overnight with the primary antibodies [Runx2, ab76956, 1:1000; osteocalcin (OCN), ab93876, 1:1000; TLR4, ab22048, 1:1000; P65, ab32536, 1:1000; p-P65, ab76302, 1:1000; Abcam, Cambridge, UK] that were used for protein detection. The membranes were subsequently incubated with secondary antibody solution for 1 h after being washed with PBS. Again, the membranes were subjected to three rinses with PBS. The protein bands were visualized using an enhanced chemiluminescence (ECL) kit (Cell Signaling Technology) in a gel imager and underwent grayscale measurement with the help of Image J software. The relative expression level of the target protein was analyzed with *β*-actin as the internal control.

### Statistical analysis

Statistical analysis was conducted using the SPSS 22.0 software. One-way ANOVA was used for comparisons among multiple groups, while t test for comparisons between two groups. All data were expressed as the mean ± standard deviation (SD). *P* < 0.05 was considered statistically significant.

## Results

### Down-regulation of miR-137 expression during osteogenic differentiation of human bone marrow mesenchymal stem cells

**Fig. 1:**
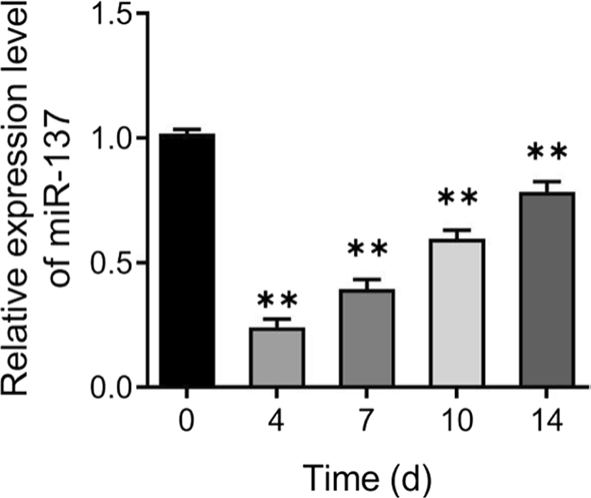
Down-regulation of miR-137 expression during osteogenic differentiation of hBMSCs. qRT-PCR analysis of miR-137 expression level in hBMSCs on days 0, 4, 7, 10, and 14 of osteogenic induction. **P < 0.01, *vs*. 0 day

**Fig. 2:**
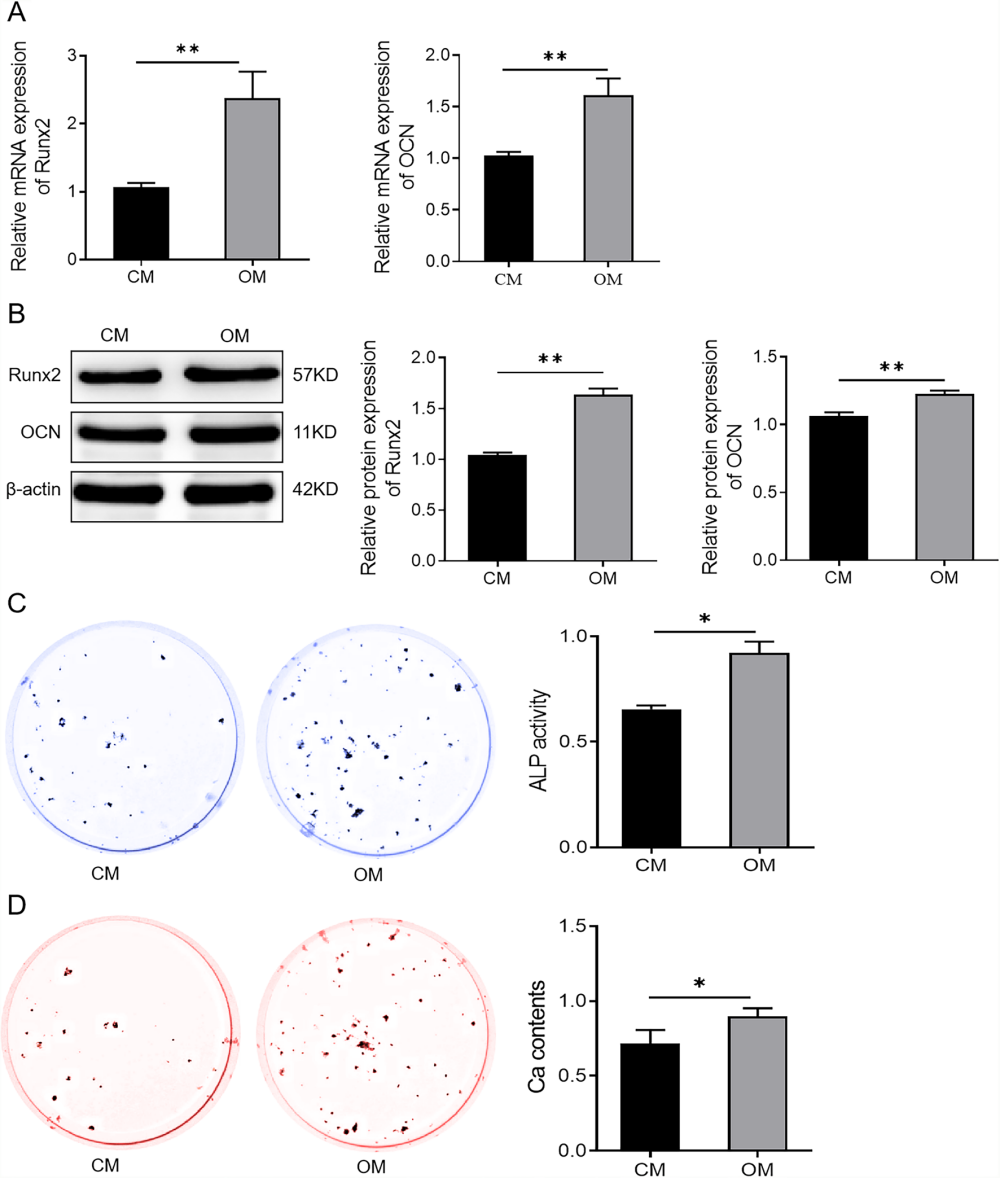
Osteogenic medium induces osteogenic differentiation of human bone marrow mesenchymal stem cells. **A** The expression levels of Runx2 and OCN in the CM and the OM groups detected by qRT-PCR. **P < 0.01. **B** Western blotting analysis of Runx2 and OCN protein levels in hBMSCs cultured in CM or OM for 7 days. **P < 0.01. **C** ALP staining to detect ALP activity of cells in the CM group and the OM group. *P < 0.05. **D** Alizarin Red staining to detect the mineralization ability of cells in the CM group and the OM group. *P < 0.05. *hBMSCs* human bone marrow mesenchymal stem cells; *Runx2* runt-related transcription factor 2; *OCN* osteocalcin; *CM* standard culture medium; *OM* osteogenic medium

To investigate the expression level of miR-137 during osteogenic differentiation of hBMSCs, hBMSCs were induced into osteoblasts and then the expression level of miR-137 was measured. Compared to day 0 of osteogenic induction, the expression level of miR-137 was significantly decreased on days 4, 7, 10, and 14 (Fig. 1A). The results of qRT-PCR and western blotting analysis showed that the expression of Runx2 and OCN in hBMSCs was significantly increased (*P* < 0.01) in the OM group compared to the CM group after the 7th day (Fig. 2A-B, Additional file 1). Subsequently, the results of ALP staining showed that the ALP activity was significantly enhanced in the cells of the OM group compared with the CM group (*P* < 0.05) (Fig. 2C). Meanwhile, the results of the Alizarin Red staining showed that the OM group exhibited notably enhanced mineralization ability of cells compared with the CM group (*P* < 0.05) (Fig. 2D).

### MiR-137 inhibits the osteogenic ability of human bone marrow mesenchymal stem cells

**Fig. 3:**
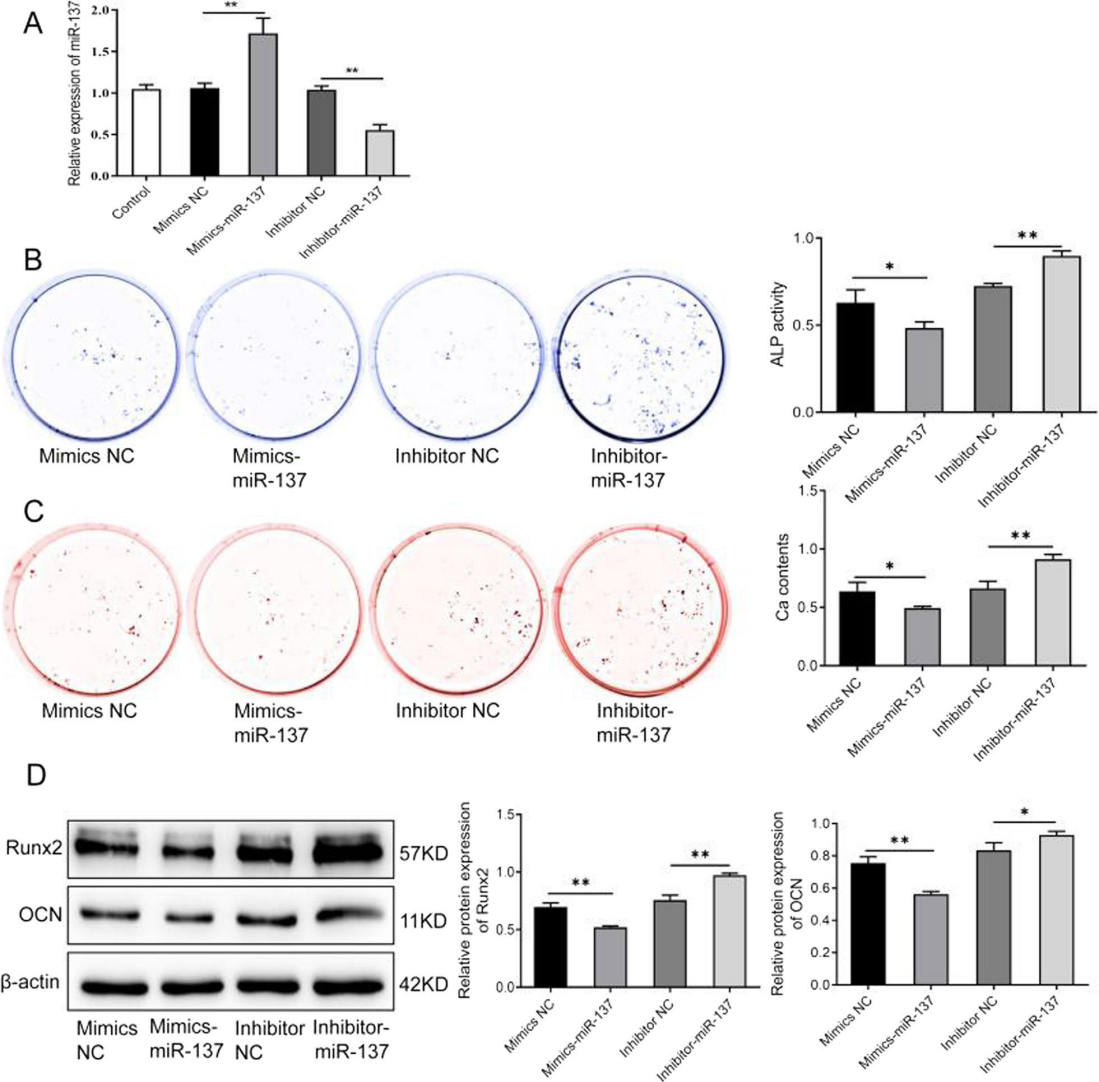
MiR-137 inhibits osteogenic differentiation of human bone marrow mesenchymal stem cells. **A** The expression level of miR-137 in mimics NC group, mimics-miR-137 group, inhibitor NC group, and inhibitor-miR-137 group detected by qRT-PCR. **B** ALP staining results of the ALP activity in mimics NC group, mimics-miR-137 group, inhibitor NC group, and inhibitor-miR-137 group. **C** Alizarin Red staining results of the mineralization ability of cells in mimics NC group, mimics-miR-137 group, inhibitor NC group, and inhibitor-miR-137 group; the calcium nodules of each group were quantitatively analyzed. **D** The protein levels of Runx2 and OCN in mimics NC group, mimics-miR-137 group, inhibitor NC group, and inhibitor-miR-137 group detected by western blotting. *P < 0.05, **P < 0.01. *NC* normal control; *ALP* alkaline phosphatase; *Runx2* runt-related transcription factor 2; *OCN* osteocalcin

We transfected hBMSCs with miR-137 mimics or inhibitors to simulate miR-137 overexpression or knockdown, respectively. The results of miRNA knockdown and overexpression are shown in Fig. 3A. It is obvious that there was no significant difference in miR-137 expression between the control group, the mimics NC group and the inhibitor NC group. However, miR-137 expression was observably increased in the mimics-miR-137 group (*P* < 0.01) and decreased in the inhibitor-miR-137 group (*P* < 0.01) compared to their NC groups. These findings indicate that miR-137 overexpression and knockdown models were successfully constructed.

Furthermore, we observed the effect of miR-137 overexpression or knockdown on the osteogenic ability and expression levels of osteogenic-related proteins in hBMSCs, in order to verify the effect of miR-137 expression on the osteogenic ability of hBMSCs. As shown in Fig. 3B, the ALP activity was obviously weakened in the mimics-miR-137 group relative to the mimics NC group. In contrast, compared to the inhibitor NC group, the ALP activity was markedly strengthened in the inhibitor-miR-137 group (*P* < 0.01). As shown in Fig. 3C, the mimics-miR-137 group had less formation of calcium nodules than the mimics NC group, whereas the inhibitor-miR-137 group showed more formation of calcium nodules than the inhibitor NC group (*P* < 0.01). Additionally, a notable decrease in the expression of the osteogenic-related proteins Runx2 and OCN was displayed in the mimics-miR-137 group, while there was an obvious increase in the two inhibitor groups (Fig. 3D, Additional file 1) (*P* < 0.05). These results suggest that miR-137 can inhibit the osteogenic ability of hBMSCs.

### MiR-137 activates the TLR4/NF-κB pathway in human bone marrow mesenchymal stem cells

**Fig. 4:**
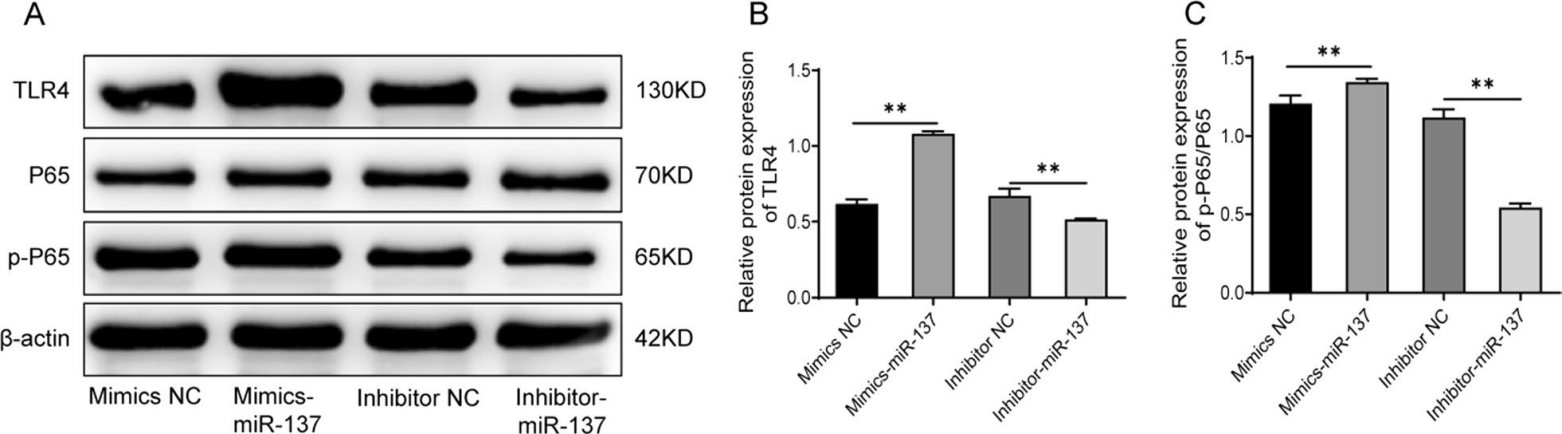
Knockdown of miR-137 suppresses the activation of the TLR4/NF-κB pathway in human bone marrow mesenchymal stem cells. **A** Western blotting analysis of TLR4, P65, and p-P65 protein levels in cells of mimics NC group, mimics-miR-137 group, inhibitor NC group, and inhibitor-miR-137 group. **B** Protein expression level of TLR4 in each group of cells. **C** The ratio of p-P65/P65 in each group of cells. * *P* < 0.05; ** *P* < 0.01. *TLR4* toll-like receptor 4, *NF-κB* nuclear factor-κB, *NC* normal control

In order to further investigate the mechanism by which miR-137 affects the osteogenic differentiation of hBMSCs, we examined the expression of TLR4/NF-κB pathway-related proteins in the cells with miR-137 overexpression or knockdown. As shown in Fig. 4A-C, and Additional file 1 overexpression of miR-137 greatly elevated the protein expression levels of TLR4 and p-P65, as well as the ratio of p-P65/P65 (*P* < 0.01). In comparison to the inhibitor NC group, the cells with inhibited miR-137 expression exhibited notably decreased protein levels of TLR4 and p-P65, as well as a reduced ratio of p-P65/P65 (*P* < 0.01). Taken together, these results indicate that miR-137 expression can activate the TLR4/NF-κB pathway in hBMSCs.

### The expression of miR-137 is negatively correlated with KDM4A expression during osteogenic differentiation of human bone marrow mesenchymal stem cells

**Fig. 5:**
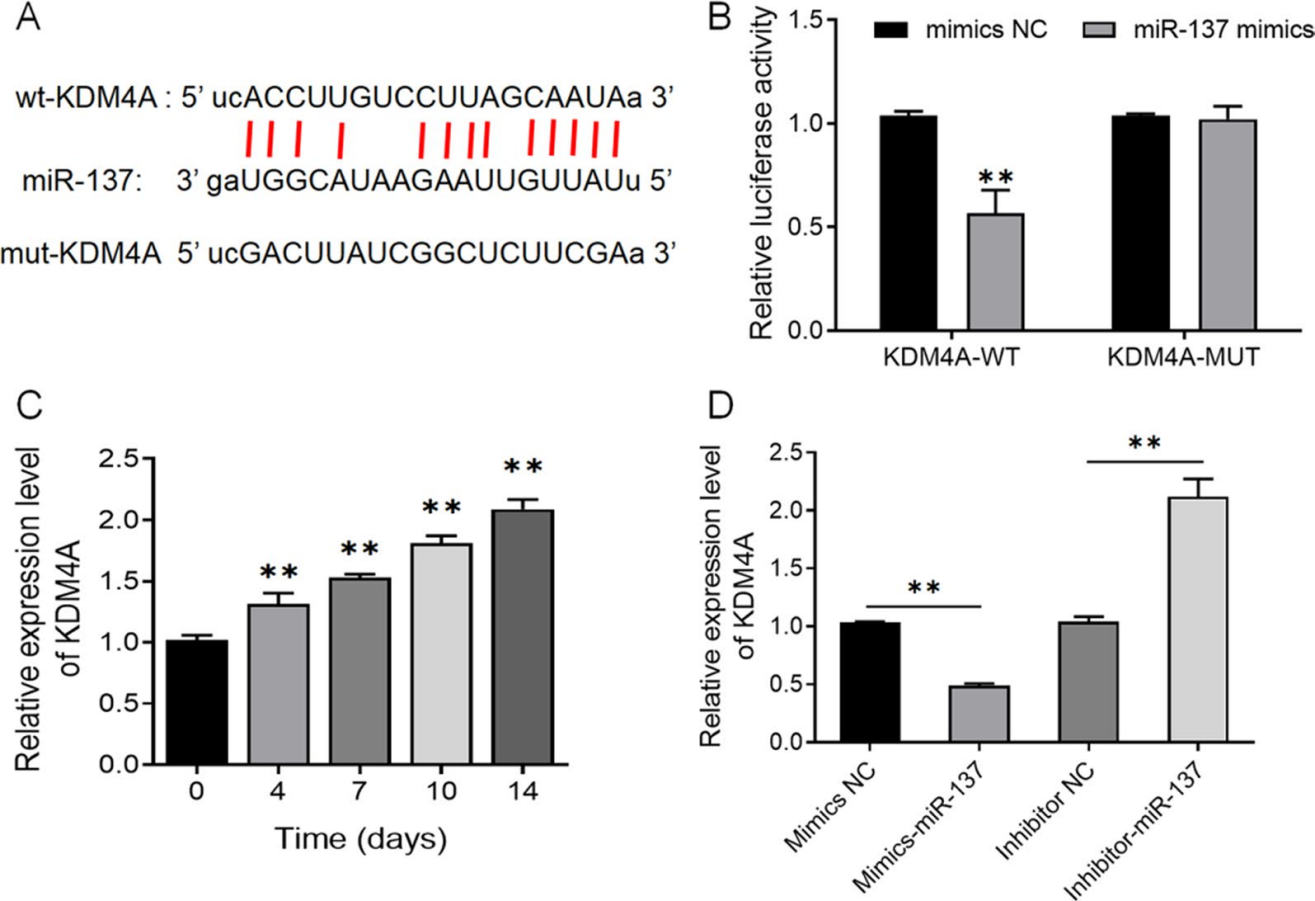
MiR-137 expression is negatively correlated with KDM4A expression. **A** Predicted target sequences of miR-137 and KDM4A. **B** The targeting relationship between miR-137 and KDM4A determined by dual-luciferase reporter assay. **P < 0.01, *vs*. mimics NC group. **C** The expression level of KDM4A in hBMSCs on days 0, 4, 7, 10, and 14 of osteogenic induction detected by qRT-PCR. **P < 0.01, *vs*. day 0. **D** The expression level of KDM4A in cells transfected with mimics NC, mimics-miR-137, inhibitor NC, and inhibitor-miR-137 detected by qRT-PCR. **P < 0.01. *KDM4A* lysine-specific demethylase 4A, *hBMSCs* human bone marrow mesenchymal stem cells; *NC* normal control

Although the above results have demonstrated that miR-137 promotes the activation of the TLR4/NF-κB pathway in hBMSCs, the mechanism by which it regulates the TLR4/NF-κB pathway is still unclear. Through bioinformatics websites, we predicted that KDM4A is a target gene of miR-137 (Fig. 5A). And according to the results of the dual-luciferase reporter assay, co-transfection of miR-137 noticeably impaired the luciferase activity of the KDM4A-WT vector but did not affect that of the KDM4A-MUT vector (Fig. 5B). In this case, we detected the expression of KDM4A in hBMSCs on days 0, 4, 7, 10, and 14 of osteogenic induction using qRT-PCR. An increase was observed in KDM4A expression (Fig. 5C), which suggests the possible involvement of KDM4A in osteogenic differentiation. The results of qRT-PCR also demonstrated that compared with the mimics NC group, the mimics-miR-137 group displayed a much higher expression level of miR-137 but a lower expression level of KDM4A, whereas compared with the inhibitor NC group, the expression level of KDM4A was significantly increased in the inhibitor-miR-137 group (Fig. 5D) (*P* < 0.01).

### MiR-137 regulates osteogenic differentiation of human bone marrow mesenchymal stem cells by targeting KDM4A

**Fig. 6:**
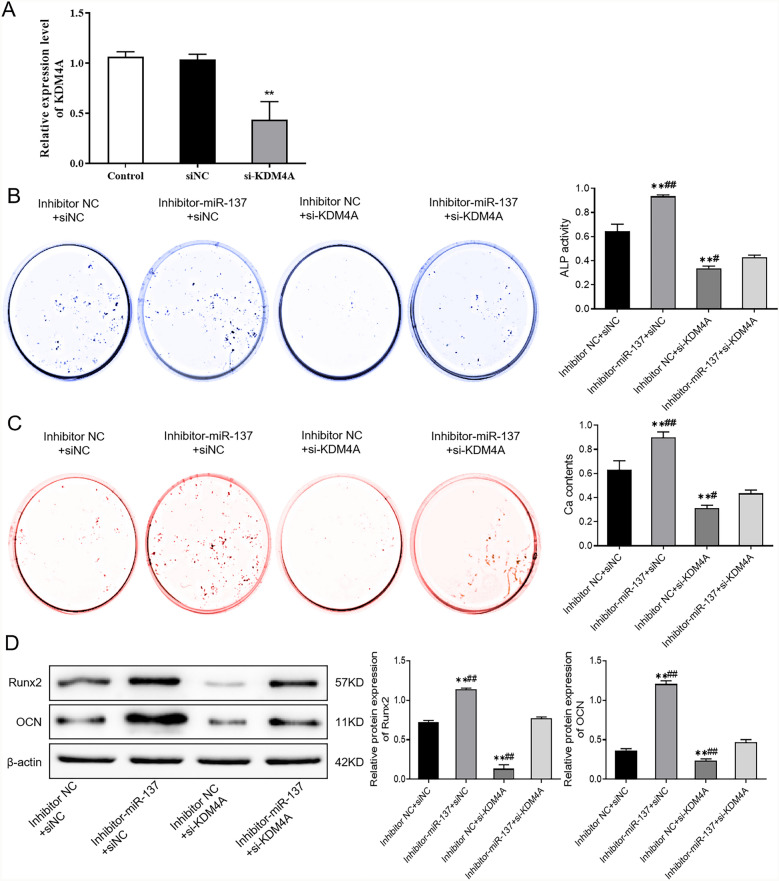
MiR-137 regulates osteogenic differentiation of hBMSCs by targeting KDM4A. **A** The expression level of KDM4A in hBMSCs after KDM4A knockdown detected by qRT-PCR. **B** ALP staining results of the activity of ALP in each group of cells. **C** Alizarin Red staining results of the mineralization ability of each group of cells. **D** The protein levels of Runx2 and OCN in each group of cells detected by western blotting. **P < 0.01, *vs*. inhibitor NC + siNC; #*P* < 0.05, ##*P* < 0.01, *vs*. inhibitor-miR-137 + si-KDM4A. *KDM4A* lysine-specific demethylase 4A, *hBMSCs* human bone marrow mesenchymal stem cells, *ALP* alkaline phosphatase, *Runx2* runt-related transcription factor 2, *OCN* osteocalcin

With the purpose of verifying the above experimental results, we transfected hBMSCs with miR-137 or KDM4A inhibitor to confirm that miR-137 can regulate TLR4/NF-κB pathway activity and promote osteogenic differentiation of hBMSCs by targeting KDM4A. In our findings, there was no significant difference in KDM4A expression between the siNC group and the control group, but the KDM4A expression was observably decreased in the si-KDM4A group (*P* < 0.01) (Fig. 6A). In addition, the activity of ALP as well as the protein levels of calcium nodules, Runx2, and OCN were much higher in the inhibitor-miR-137 + siNC group than in the inhibitor NC + siNC group, while those levels were reduced after knockout of si-KDM4A (*P* < 0.01). However, the activity of ALP and the protein levels of calcium nodules, Runx2, and OCN in the cells with knockdown of miR-137 and KDM4A were apparently lower than those in the inhibitor-miR-137 + siNC group, but much higher than those in the inhibitor NC + si-KDM4A group (*P* < 0.01) (Fig. 6B–D, Additional file 1). In a nutshell, KDM4A can promote osteogenic differentiation in hBMSCs, and the osteogenic ability reduced by KDM4A inhibition can be partially restored after knocking down miR-137 expression.

### MiR-137 activates the TLR4/NF-κB pathway activity by targeting KDM4A

**Fig. 7:**
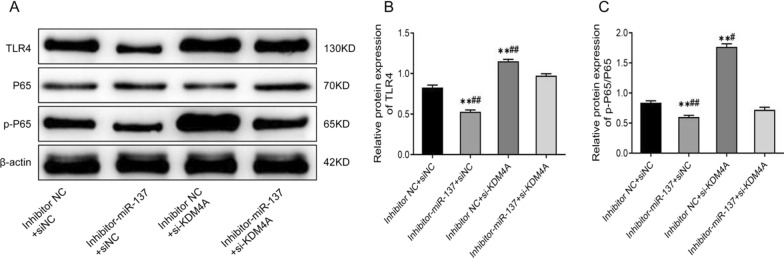
MiR-137 activates TLR4/NF-κB pathway activity by targeting KDM4A. **A**–**C** Western blotting analysis results of the protein levels of TLR4, P65, and p-P65 in each group of cells. **P < 0.01, *vs*. inhibitor NC + siNC group; #*P* < 0.05, ##*P* < 0.01, *vs*. inhibitor-miR-137 + si-KDM4A group

As shown in Fig. 7A–C and Additional file 1 the protein expression of TLR4 and p-P65 was remarkably lowered, and the p-P65/P65 ratio was significantly reduced in the inhibitor-miR-137 + siNC group compared to the inhibitor NC + siNC group. The protein levels of TLR4 and p-P65 were considerably increased in the inhibitor NC + si-KDM4A group, as well as the p-P65/P65 ratio (*P* < 0.01). More importantly, the protein levels of TLR4 and p-P65, as well as the p-P65/P65 ratio in the inhibitor-miR-137 + si-KDM4A group, were quite higher than those in the inhibitor-miR-137 + siNC group but much lower than those in the inhibitor NC + si-KDM4A group (*P* < 0.01). All in all, inhibition of miR-137 can partially suppress the TLR4/NF-κB pathway activity enhanced by KDM4A inhibition.

## Discussion

Osteoporosis is a chronic disease that has a negative impact on quality of life of its patients. In terms of its incidence, it is estimated that over 75 million people worldwide have osteoporosis [3]. Previous studies have shown that the development of osteoporosis is related to various factors, including genetic and environmental factors, most of which affect the differentiation and activity of osteoblasts and osteoclasts [1]. In recent years, research in the field of bone formation has also described that miR-137 can regulate the differentiation of osteoblasts or osteoclasts. Moreover, lncRNA FTX can reduce the generation of osteoclasts and inhibit osteoblast differentiation by targeting miR-137 [23]. However, the dysregulation of miRNA-137 puts people at risk for osteoporotic fracture because it inhibits ALP activity and expression via suppressing the expression of leucine-rich repeat-containing G-protein-coupled receptor 4 [24]. Therefore, we speculated that miR-137 may worsen the progression of osteoporosis through a certain medium.

Firstly, we verified that miR-137 can prevent hBMSCs from differentiating into osteoblasts through simulating overexpression and knockdown of miR-137. Increased ALP activity and calcium salt deposition are key markers of cell osteogenic differentiation. The emergence of ALP during the initial stages of osteoblast differentiation can accelerate the mineralization of the bone matrix [25]. Extracellular calcium salt deposition is an essential sign of the late stage of osteoblast differentiation [26]. The expression of osteogenic-related genes is also an indicator of the cell capacity for osteogenic differentiation. Osteoporosis is caused by an imbalance between bone resorption and bone formation. Because osteoclasts play a more potent function than osteoblasts, there will eventually be less bone density and bone loss. RUNX2, an osteogenic-related gene, is one of the most important transcription factors involved in the osteogenic differentiation of hBMSCs [27]. RUNX2 regulates the gene transcription of key proteins related to osteogenic differentiation, which assists hBMSCs in differentiating into osteoblasts [28]. Osteocalcin (OCN) is another important indicator in the process of osteogenesis. As a constituent protein of bone matrix, OCN is primarily synthesized by osteoblasts and is crucial in controlling bone calcium metabolism [29]. In this study, we found that miR-137 inhibited the activity of ALP, the formation of calcium nodules, and the expression of Runx2 and OCN, indicating that miR-137 can inhibit the osteogenic ability of hBMSCs.

Subsequently, whether miR-137 could affect osteogenic differentiation by regulating the TLR4/NF-κB pathway was investigated. As revealed in previous studies, inflammation and inflammatory factors play a crucial role in the occurrence, development, and prognosis of major diseases such as autoimmune and degenerative diseases. Toll-like receptors (TLRs) -mediated inflammatory responses are vital in bone formation system [30]. Especially, the signal transduction pathway of TLR4 can activate NF-κB continuously, thus promoting the transcription of various inflammatory factors and resulting in a cascade of inflammatory responses. NF-κB, a major transcription factor in inflammatory response, regulates the differentiation of both osteoblasts and osteoclasts and maintains bone homeostasis [31]. Our results showed that miR-137 significantly increased the protein expression levels of TLR4 and p65, which indicates that miR-137 may regulate osteogenic differentiation by activating the TLR4/NF-κB signaling pathway in hBMSCs.

Through prediction of the target mRNA of miR-137, KDM4A was speculated to be a potential mediator of miR-137 in the activation the TLR4/NF-κB pathway. In order to validate the speculation, we performed knockdown experiments on KDM4A. The control of mesenchymal stem cell (MSC) lineage development depends critically on the epigenetic mechanism mediated by histone demethylases (KDMs). In this study, we identified KDM4A as a novel epigenetic regulator of osteogenic and adipogenic differentiation. It follows that miR-137 inhibits the osteogenesis in hBMSCs, and that miR-137 enhances TLR4/NF-κB pathway activity by targeting KDM4A. In this case, MSC osteogenic differentiation is inhibited and osteoporosis is exacerbated. Overall, miR-137 may affect the osteogenic ability of hBMSCs through the activation of the TLR4/NF-κB pathway.

Further validation of miR-137expression in clinical bone marrow tissue could provide additional evidence of its role in osteoporosis. The outcomes of this study, however, were not clinically validated because it lacked clinical samples. Apart from that, additional mechanistic testing in animal models can provide a more comprehensive explanation for the strong association between miR-137 expression level and the exacerbation of osteoporosis. Therefore, further in vivo experiments and clinical sample data are needed to support the existing results. Since miR-137 has been shown to be closely related to cell proliferation and differentiation, the bone formation-related pathways regulated by miR-137 may not be limited to TLR4/NF-κB. In our future studies, it is anticipated that the role of miR-137 in osteoporosis will be further investigated.

## Conclusion

Taken together, miR-137 can activate TLR4/NF-κB pathway, inhibit osteogenic differentiation, and thus exacerbate osteoporosis in the osteogenesis of hBMSCs. Accordingly, miR-137 may be a potential target for the treatment of osteoporosis, and further research is required to explore its specific mechanism.

## Supplementary Information

**Additional file 1. **Protein expression levels of Runx2, OCN, and TLR4/NF-κB determined by Western blot

## Data Availability

The data that support the findings of this study are available from the authors upon reasonable request.
