# Correction: MiR-137 promotes TLR4/NF-κB pathway activity through targeting KDM4 A, inhibits osteogenic diferentiation of human bone marrow mesenchymal stem cells and aggravates osteoporosis

**DOI:** 10.1186/s13018-025-05844-7

**Published:** 2025-05-22

**Authors:** Ying-feng Yu, Pei-quan Yao, Zhi-kun Wang, Wen-wei Xie

**Affiliations:** Department of Orthopedics, Songshan Lake Central Hospital of Dongguan City, Dongguan, Guangdong China

**Correction: J Orthop Surg Res, (2023)18:444**

**https://doi.org/10.1186/s13018-023–03918-y**

In this article Figs. 2 and 6 appeared incorrectly and have now been corrected in the original publication. For completeness and transparency, the old incorrect versions are displayed below.

Incorrect Fig. 2

**Fig. 2:**
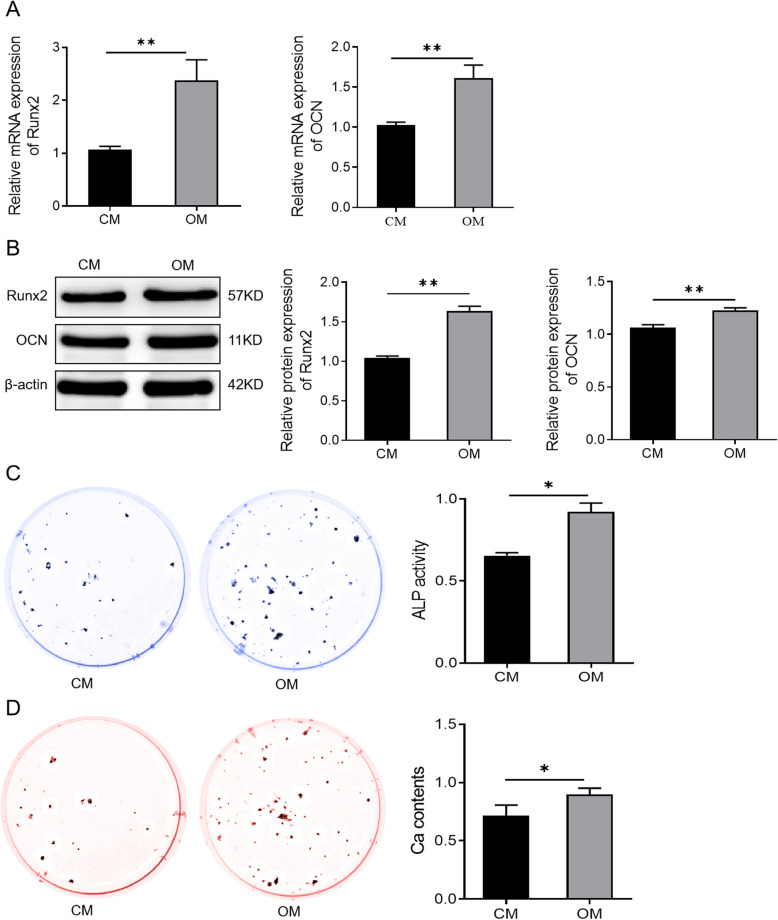
Osteogenic medium induces osteogenic differentiation of human bone marrow mesenchymal stem cells. **A** The expression levels of Runx2 and OCN in the CM and the OM groups detected by qRT-PCR. **P < 0.01. **B** Western blotting analysis of Runx2 and OCN protein levels in hBMSCs cultured in CM or OM for 7 days. **P < 0.01. **C** ALP staining to detect ALP activity of cells in the CM group and the OM group. *P < 0.05. **D** Alizarin Red staining to detect the mineralization ability of cells in the CM group and the OM group. *P < 0.05. *hBMSCs* human bone marrow mesenchymal stem cells; *Runx2* runt-related transcription factor 2; *OCN* osteocalcin; *CM* standard culture medium; *OM* osteogenic medium

Corrected Fig. 2

**Fig. 2:**
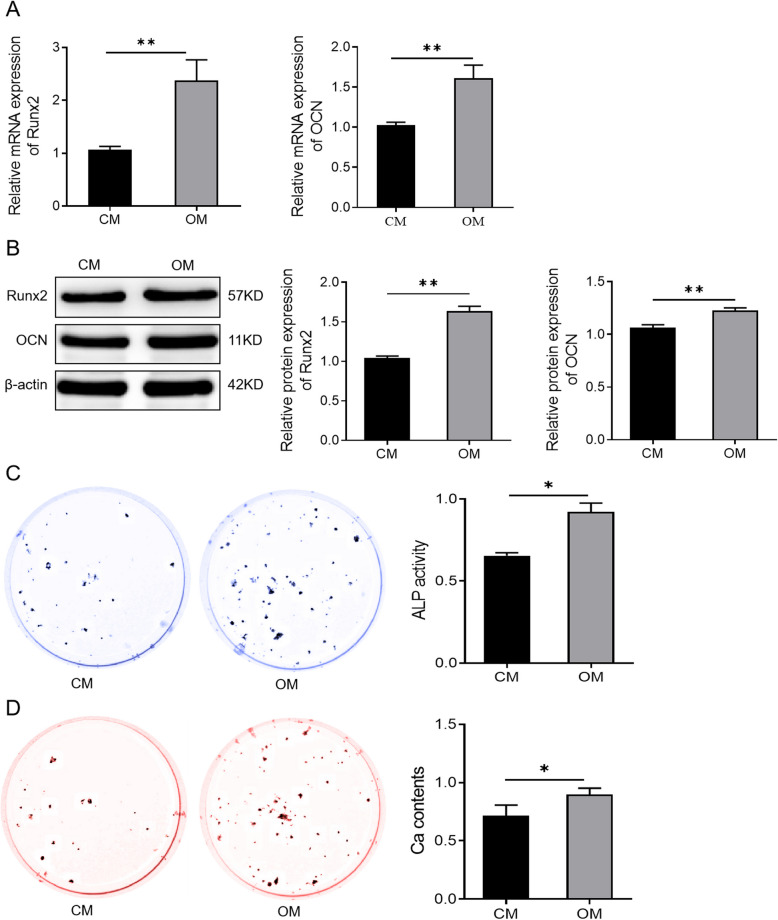
Osteogenic medium induces osteogenic differentiation of human bone marrow mesenchymal stem cells. **A** The expression levels of Runx2 and OCN in the CM and the OM groups detected by qRT-PCR. **P < 0.01. **B** Western blotting analysis of Runx2 and OCN protein levels in hBMSCs cultured in CM or OM for 7 days. **P < 0.01. **C** ALP staining to detect ALP activity of cells in the CM group and the OM group. *P < 0.05. **D** Alizarin Red staining to detect the mineralization ability of cells in the CM group and the OM group. *P < 0.05. *hBMSCs* human bone marrow mesenchymal stem cells; *Runx2* runt-related transcription factor 2; *OCN* osteocalcin; *CM* standard culture medium; *OM* osteogenic medium

Incorrect Fig. 6

**Fig. 6:**
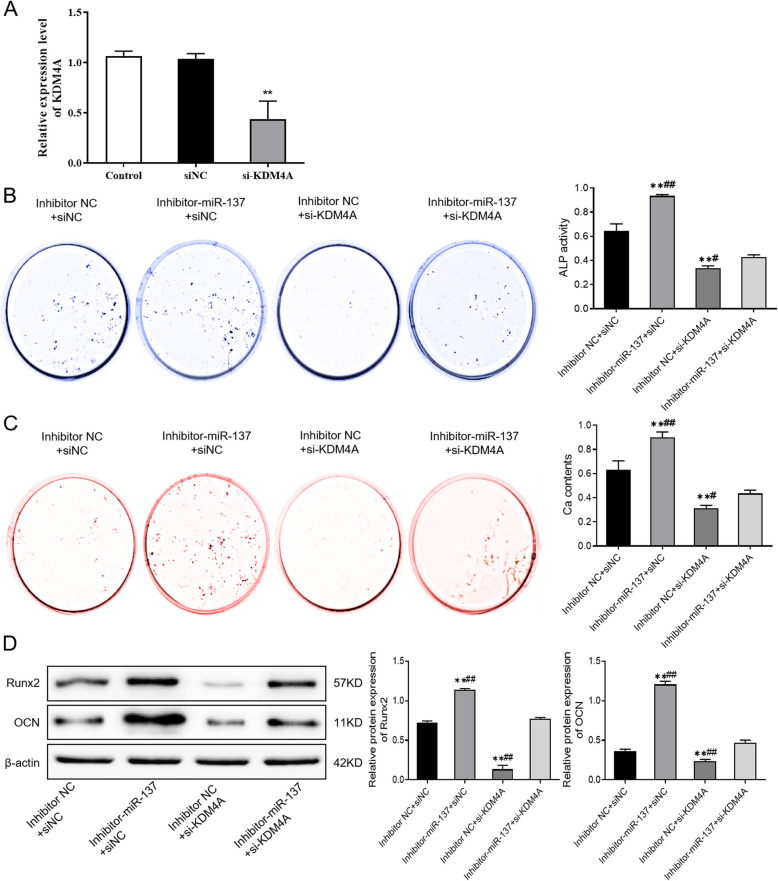
MiR-137 regulates osteogenic differentiation of hBMSCs by targeting KDM4A. **A** The expression level of KDM4A in hBMSCs after KDM4A knockdown detected by qRT-PCR. **B** ALP staining results of the activity of ALP in each group of cells. **C** Alizarin Red staining results of the mineralization ability of each group of cells. **D** The protein levels of Runx2 and OCN in each group of cells detected by western blotting. **P < 0.01, *vs*. inhibitor NC + siNC; #*P* < 0.05, ##*P* < 0.01, *vs*. inhibitor-miR-137 + si-KDM4A. *KDM4A* lysine-specific demethylase 4A, *hBMSCs* human bone marrow mesenchymal stem cells, *ALP* alkaline phosphatase, *Runx2* runt-related transcription factor 2, *OCN* osteocalcin

Correct Fig. 6

**Fig. 6:**
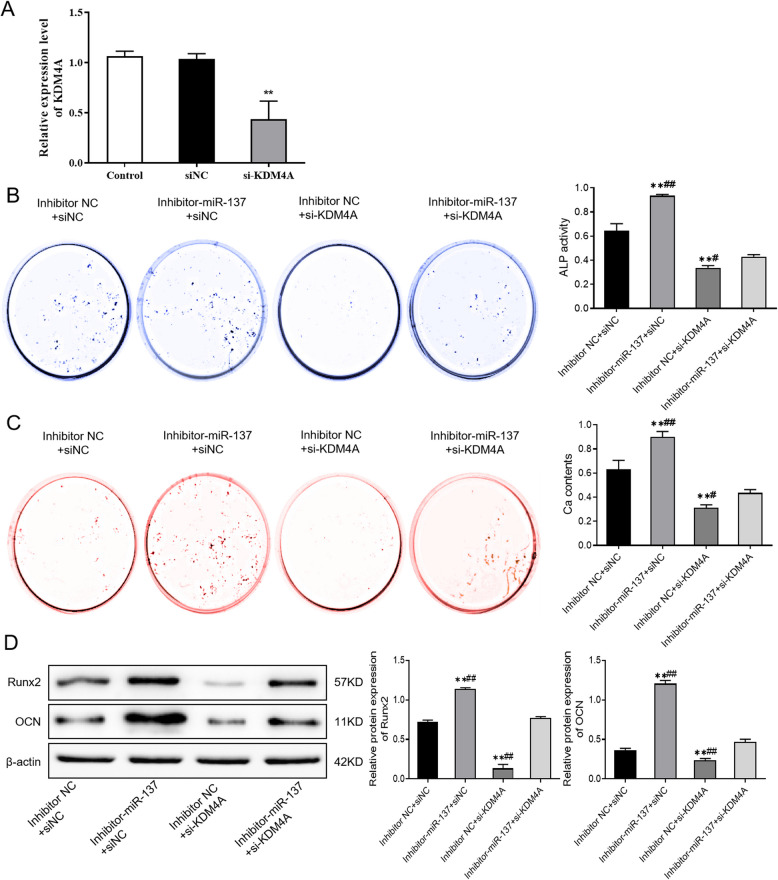
MiR-137 regulates osteogenic differentiation of hBMSCs by targeting KDM4A. **A** The expression level of KDM4A in hBMSCs after KDM4A knockdown detected by qRT-PCR. **B** ALP staining results of the activity of ALP in each group of cells. **C** Alizarin Red staining results of the mineralization ability of each group of cells. **D** The protein levels of Runx2 and OCN in each group of cells detected by western blotting. **P < 0.01, *vs*. inhibitor NC + siNC; #*P* < 0.05, ##*P* < 0.01, *vs*. inhibitor-miR-137 + si-KDM4A. *KDM4A* lysine-specific demethylase 4A, *hBMSCs* human bone marrow mesenchymal stem cells, *ALP* alkaline phosphatase, *Runx2* runt-related transcription factor 2, *OCN* osteocalcin

The original article has been corrected.

