# Supplementary material for: MiR-137 promotes TLR4/NF-κB pathway activity through targeting KDM4A, inhibits osteogenic differentiation of human bone marrow mesenchymal stem cells and aggravates osteoporosis
Source: J Orthop Surg Res. 2023 Jun 21;18:444. doi: 10.1186/s13018-023-03918-y (PMC10286393; doi:10.1186/s13018-023-03918-y)
Supplement: Supplementary file 1 — Additional file 1. Protein expression levels of Runx2, OCN, and TLR4/NF-κB determined by Western blot [file 13018_2023_3918_MOESM1_ESM.pdf]

Figure 2B

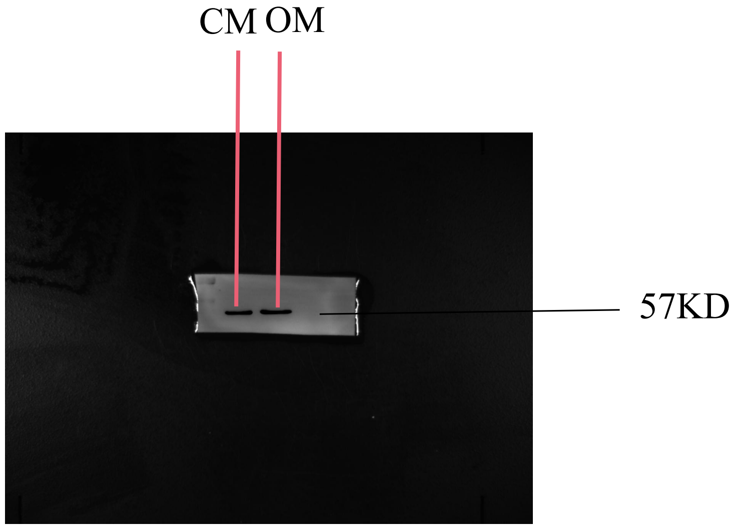

Runx2

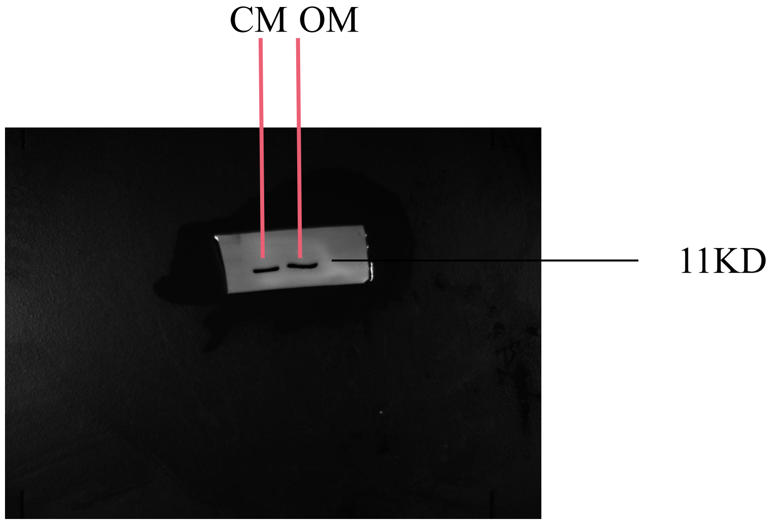

OCN

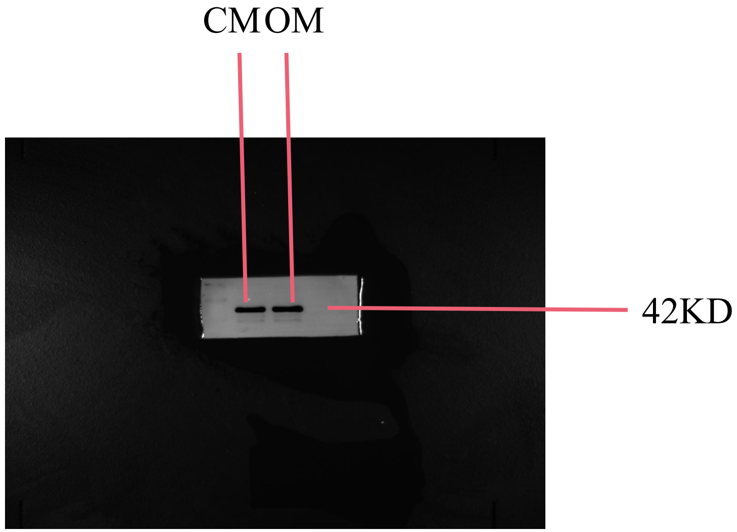

β-actin

Figure 3D

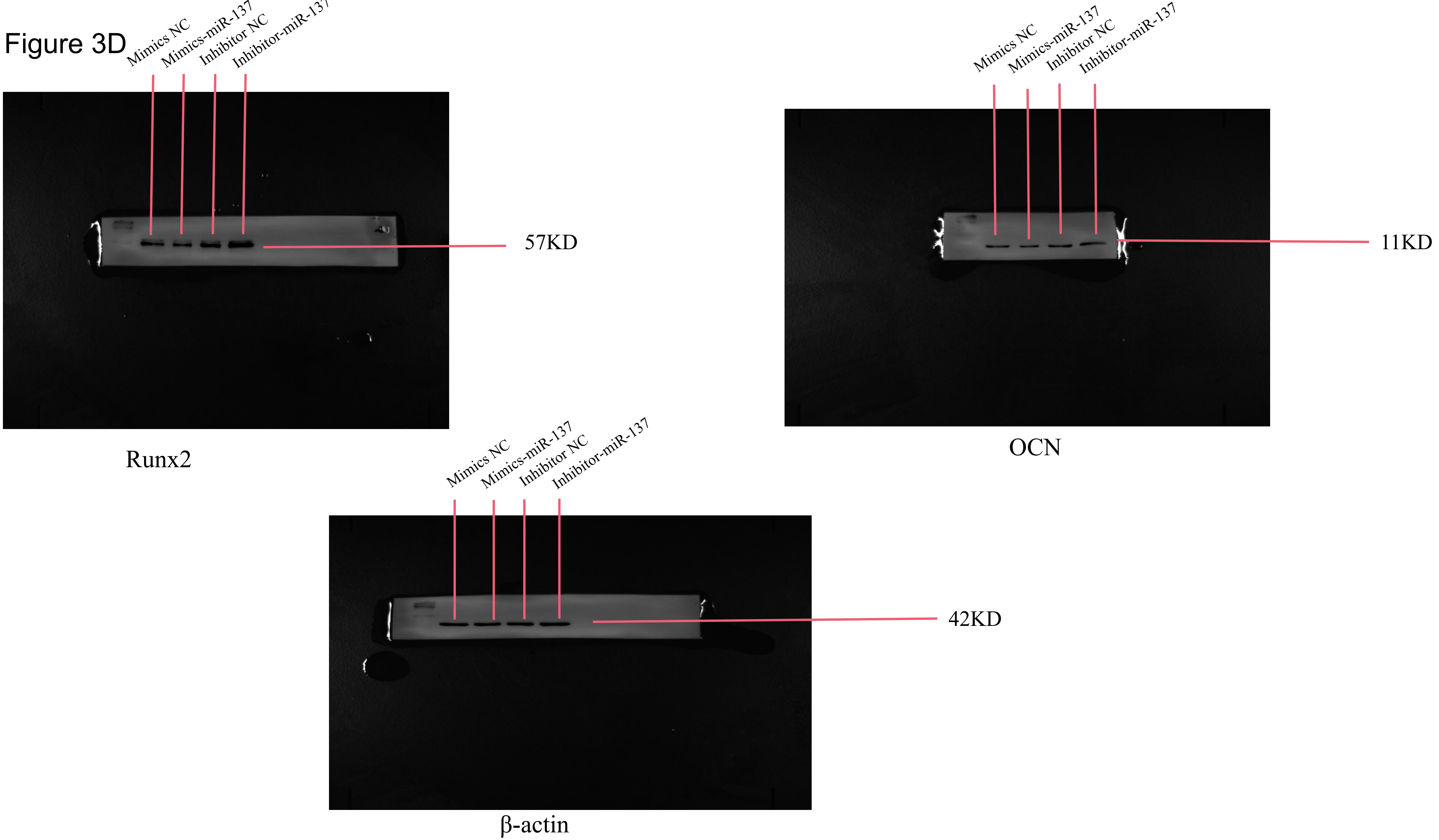
Mimics NC
Mimics-miR-137
Inhibitor NC
Inhibitor-miR-137
57KD
Runx2
Mimics NC
Mimics-miR-137
Inhibitor NC
Inhibitor-miR-137
11KD
OCN
Mimics NC
Mimics-miR-137
Inhibitor NC
Inhibitor-miR-137
42KD
β-actin

Figure 4A

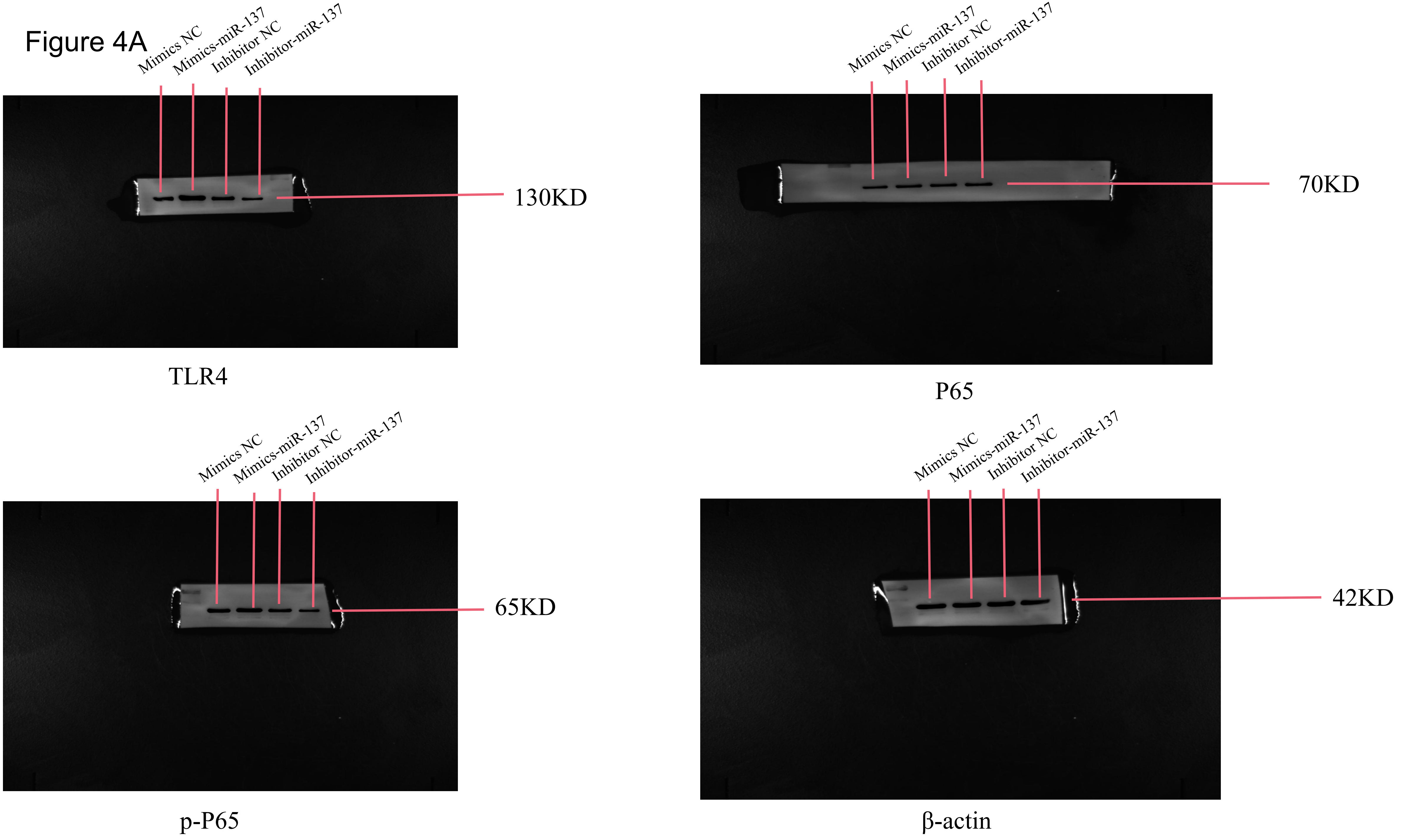
Mimics NC
Mimics-miR-137
Inhibitor NC
Inhibitor-miR-137
130KD
TLR4
Mimics NC
Mimics-miR-137
Inhibitor NC
Inhibitor-miR-137
70KD
P65
Mimics NC
Mimics-miR-137
Inhibitor NC
Inhibitor-miR-137
65KD
p-P65
Mimics NC
Mimics-miR-137
Inhibitor NC
Inhibitor-miR-137
42KD
β-actin

Figure 6D

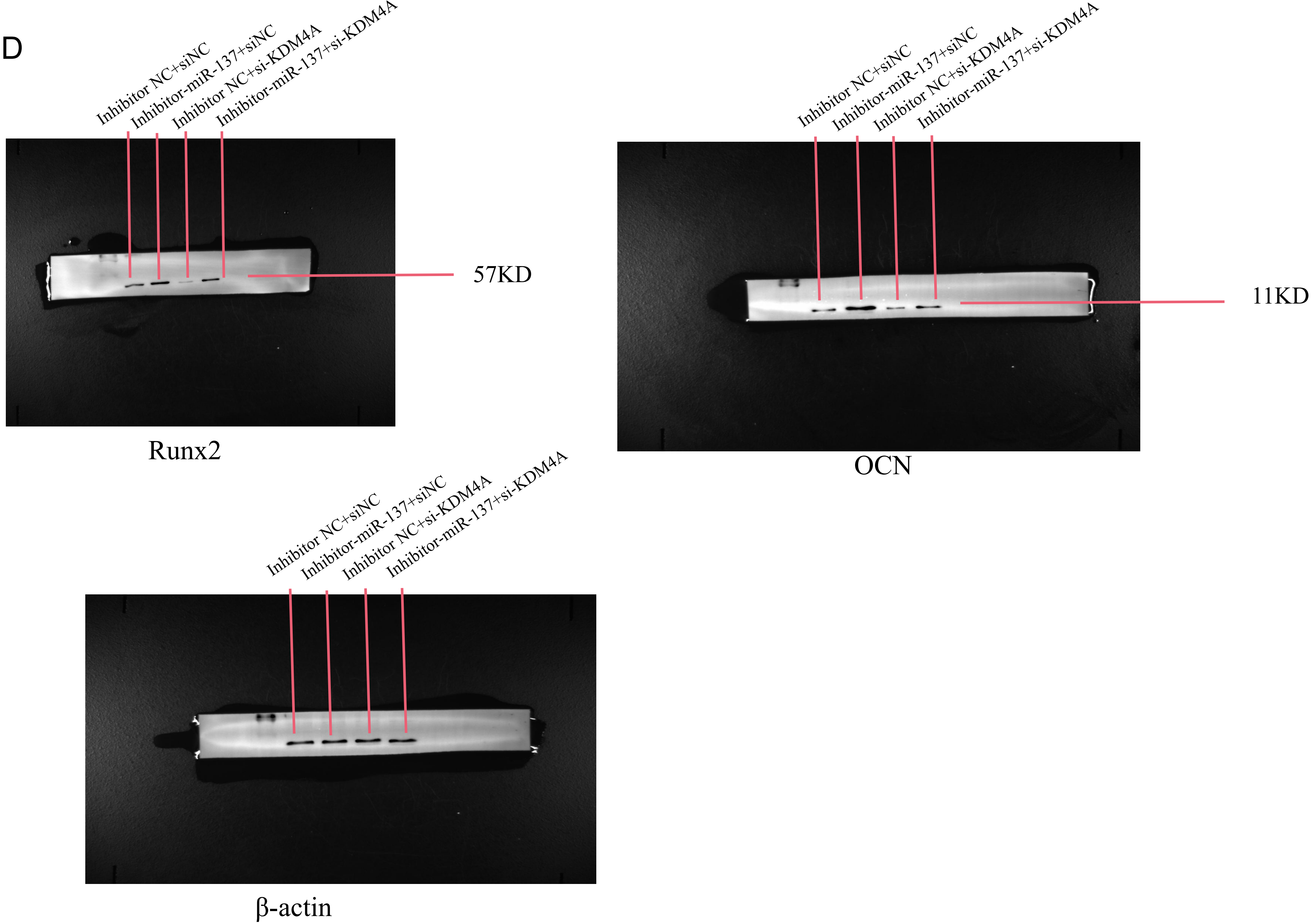
Inhibitor NC+siNC
Inhibitor-miR-137+siNC
Inhibitor NC+si-KDM4A
Inhibitor-miR-137+si-KDM4A
57KD
Runx2
Inhibitor NC+siNC
Inhibitor-miR-137+siNC
Inhibitor NC+si-KDM4A
Inhibitor-miR-137+si-KDM4A
11KD
OCN
Inhibitor NC+siNC
Inhibitor-miR-137+siNC
Inhibitor NC+si-KDM4A
Inhibitor-miR-137+si-KDM4A
β-actin

Figure 7A

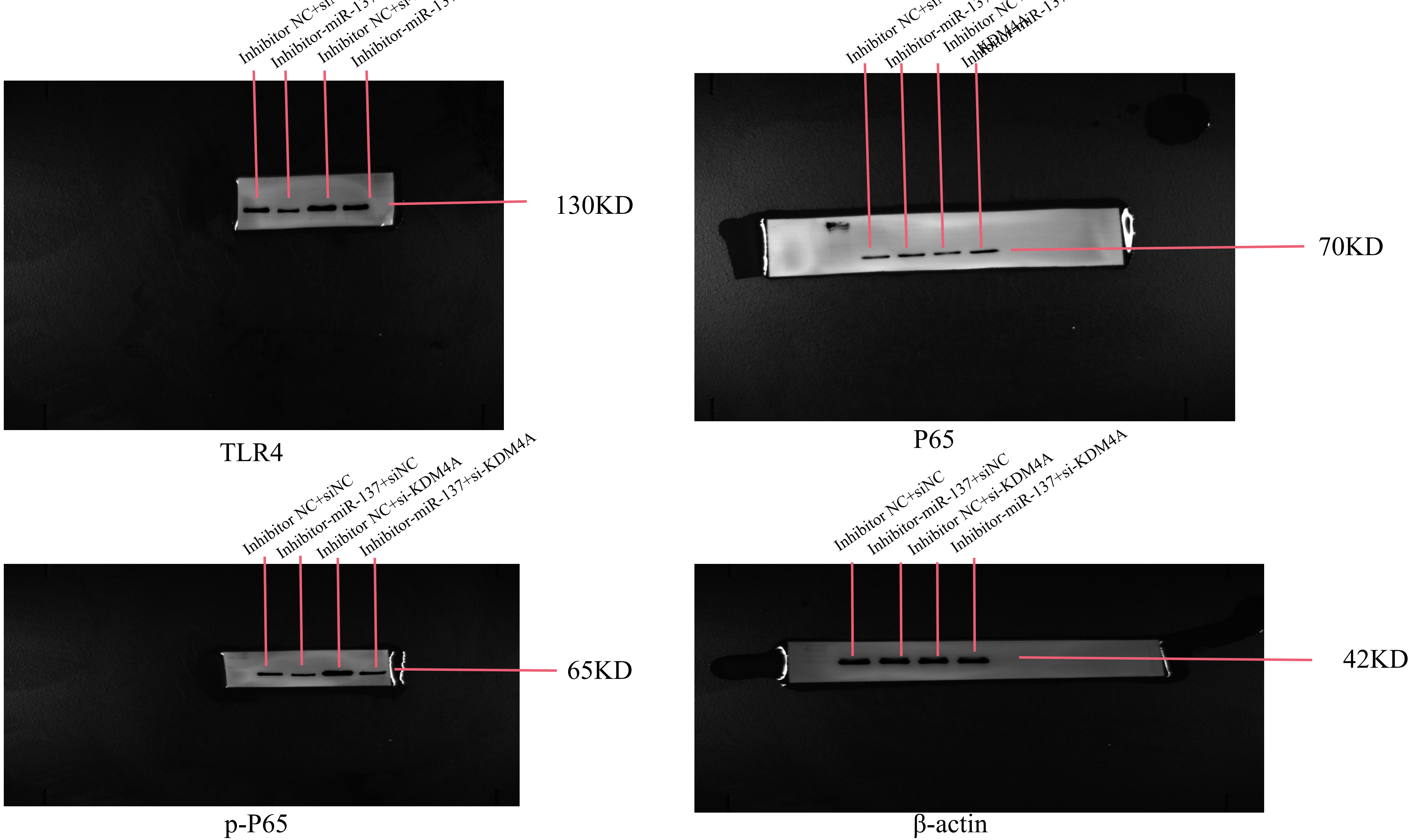
Inhibitor NC+siNC
Inhibitor-miR-137+siNC
Inhibitor NC+si-KDM4A
Inhibitor-miR-137+si-KDM4A
130KD
TLR4
Inhibitor NC+siNC
Inhibitor-miR-137+siNC
Inhibitor NC+si-KDM4A
Inhibitor-miR-137+si-KDM4A
70KD
P65
Inhibitor NC+siNC
Inhibitor-miR-137+siNC
Inhibitor NC+si-KDM4A
Inhibitor-miR-137+si-KDM4A
65KD
p-P65
Inhibitor NC+siNC
Inhibitor-miR-137+siNC
Inhibitor NC+si-KDM4A
Inhibitor-miR-137+si-KDM4A
42KD
β-actin
